# The Draft Genome of a Hydrogen-producing Cyanobacterium, *Arthrospira platensis* NIES-46

**DOI:** 10.7150/jgen.38149

**Published:** 2019-09-18

**Authors:** Shigekatsu Suzuki, Haruyo Yamaguchi, Masanobu Kawachi

**Affiliations:** Center for Biology and Environmental Studies, National Institute for Environmental Studies, 16-2 Onogawa, Tsukuba, Ibaraki, Japan.

**Keywords:** *Arthrospira platensis* NIES-46, filamentous cyanobacterium, genome, hydrogen production, phylogeny, *Spirulina*

## Abstract

*Arthrospira* is an economically important cyanobacterium that contains many useful products, including proteins, vitamins, lipids, and pigments, and it is distributed in several alkaline soda lakes. *Arthrospira platensis* NIES-46 produces large amounts of hydrogen. In this study, we sequenced the NIES-46 draft genome and performed comparative analyses among *Arthrospira* species to elucidate the genomic background of this strain. The genome consists of 5.7 Mbp with a GC% of 44.5% and encodes 5,008 proteins. Our phylogenetic analysis using multiple orthologous proteins shows that *Arthrospira* is divided into two clades and that NIES-46 is closely related to *A. platensis* NIES-39. The genome structure and protein functions are highly conserved between *A. platensis* NIES-39 and NIES-46, suggesting that these two strains have recently diverged. Genes involved in hydrogen production are well-conserved among *Arthrospira* species, indicating conserved abilities to produce hydrogen.

## Introduction

The filamentous, non-N_2_-fixing cyanobacterium *Arthrospira* is an economically important organism that contains a large number of proteins, vitamins, lipids, and pigments, and it is widely used in human and animal food 1. The natural habitat of *Arthrospira* is alkaline soda lakes such as Lake Chad in Chad, Lake Texcoco in Mexico, and Lake Chenghai in China; therefore, *Arthrospira* can tolerate high pH and salt environments 2. These are useful characteristics for mass-cultivation in open ponds because these conditions can be toxic for contaminating species and predators.

In addition to some nutrients and other useful products, *Arthrospira* can produce hydrogen via a bidirectional [NiFe]-hydrogenase, an enzyme that catalyses the reversible reduction of protons to H_2_ coupled to NAD^+^ regeneration 3. This enzyme is composed of five subunits encoded by the *hox* gene cluster (*hoxEFUHY*). Accessory proteins (encoded by the *hypABCDEF* gene cluster) are required for the maturation of the active site within the catalytic cluster of the hydrogenase subunit 3. Several cyanobacterial genomes possess the *hox* and *hyp* gene clusters 3. In particular, *Arthrospira maxima* is one of the most efficient hydrogen-producing cyanobacterium 4,5, and *Arthrospira platensis* also produces large amounts of hydrogen 6. In *A. platensis* NIES-46, detailed physiology and culture conditions for hydrogen production have been reported 7. NIES-46 produces hydrogen under dark anaerobic conditions and is enhanced by nitrogen-starvation. However, the genome sequence of NIES-46 is unavailable.

To date, the complete genomes of nine *Arthrospira* strains have been sequenced, including *A. maxima*, *A. platensis*, and unidentified *Arthrospira* species (https://www.ncbi.nlm.nih.gov/genomes/GenomesGroup.cgi?taxid=35823). These genomes have many repetitive sequences (e.g., group II introns) and a wide variety of restriction-modification systems 8-10. The repetitive sequences underlie the high genome plasticity observed in this genus 9. *Arthrospira* genomes also encode NapA-type Na+/H+ antiporter homologs, which can be involved in salt tolerance in alkaline habitats 11. The *Arthrospira* species with available genomes were sampled from lakes or ponds in Africa (NIES-39 and UTEX2342), Europe (Paraca and O9.13F), and Asia (TJSD091 and TJSD092), while the sampling locations of two strains (PCC8005 and PCC9438) are unknown. No genomes of *Arthrospira* strains sampled on the American continent have been sequenced, and such an undertaking could be interesting for gaining more insight into *Arthrospira* evolution based on having a more complete picture of its global distribution. In this study, we sequenced the genome of *A. platensis* NIES-46, which was sampled from Lake Texcoco in Mexico.

## Material and Methods

*Arthrospira platensis* NIES-46 was maintained in 10 mL SOT medium 12 at 20ºC under a 10 h:14 h light:dark cycle. The cells were collected via gentle centrifugation. DNA was extracted using NucleoBond Buffer Set III and NucleoBond AXG 100 (Macherey-Nagel, Düren, Germany), following the manufacturer's protocol. The DNA was sheared into fragments of about 550 bp using a Covaris M220 ultrasonicator (Covaris, Woburn, MA). The DNA library for the MiSeq sequencing was prepared using the NEBNext Ultra II DNA Library Prep Kit for Illumina (New England Biolabs, Ipswich, MA). Paired-end reads (257,288,461 bp) were obtained on the MiSeq platform (Illumina, San Diego, CA) with the 600-cycle MiSeq Reagent Kit v3. The raw reads were trimmed, assembled, and error-corrected using Trimmomatic 0.38 13, SPAdes v3.11.1 14, and Pilon v1.22 15, respectively, all of which were implemented in Shovill v1.0.4 (https://github.com/tseemann/shovill). After removing the short reads (< 200 bp), a gene model construction was generated using the DFAST legacy server 16. Functional annotation was performed using the EggNOG web server 17. The draft genome was deposited to the DDBJ/Genbank/ENA repositories with the accession numbers BIMW01000001-BIMW01000343. For phylogenetic analysis, 126 conservative orthologs were used. *Lyngbya sp*. PCC8106 and *Trichodesmium erythraeum* IMS101 were used as an out group based on a previous report 8. The orthologs were determined via reciprocal BLAST best-hit analyses with the following cut-offs: similarity >80% and HSP coverage >80%. Model tests and maximum-likelihood analyses with 200 non-parametric bootstrap replications were performed via IQ-TREE 1.7 25.

## Results and Discussion

**Figure 1:**
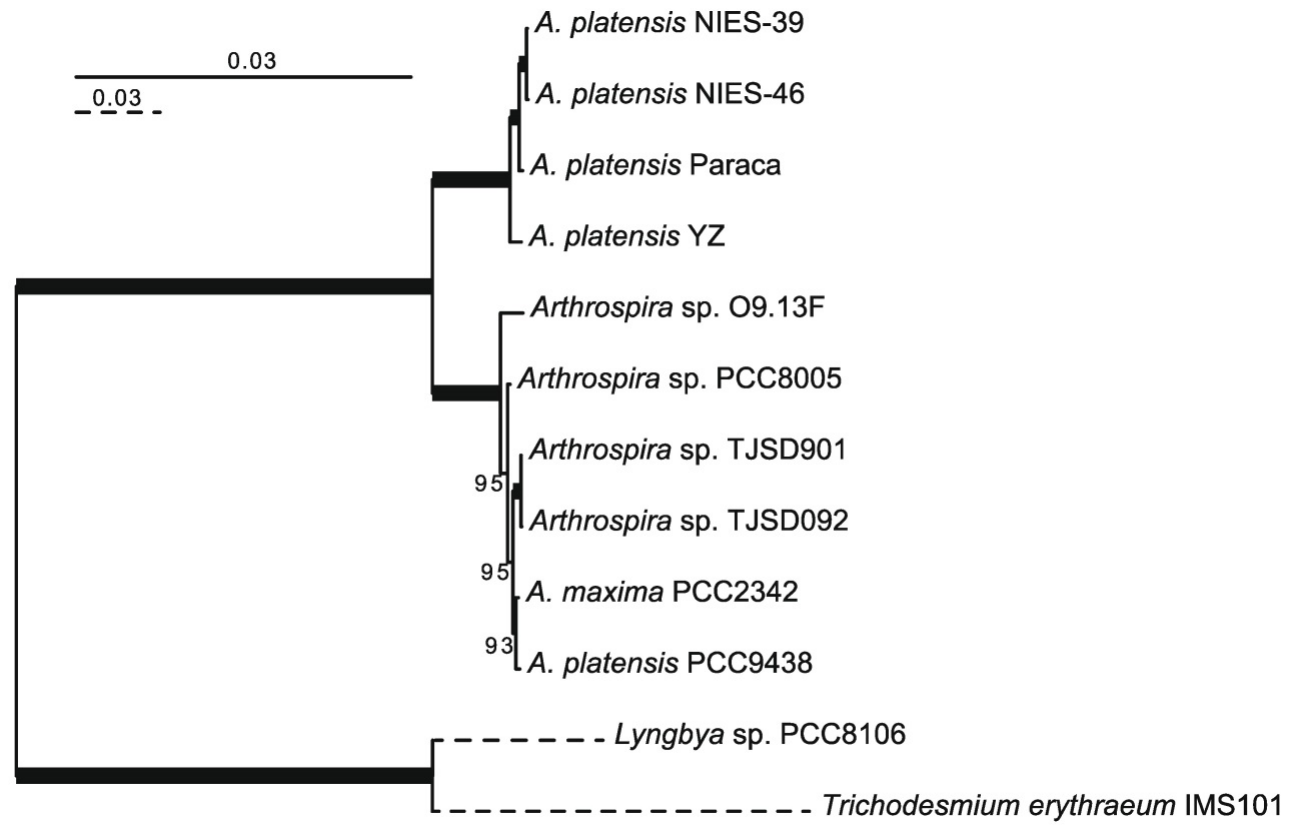
** Phylogenetic tree of *Arthrospira* species.** A maximum-likelihood tree was inferred using 126 orthologous proteins. The bootstrap percentages (BPs) are indicated on the nodes. The bold lines represent 100% BP. The dotted branches are shown in quarter-length.

The NIES-46 draft genome is composed of 343 contigs and 5,728,646 bp. The genome completeness is 100% based on a comparison with cyanobacterial marker genes using checkM analysis 19. The GC% of the genome is 44.5%, and it encodes 5,008 proteins, four rRNAs, 38 tRNAs, and eight CRISPR clusters (Table 1). To infer the phylogenetic relationships between NIES-46 and other *Arthrospira* strains, we performed a phylogenetic analysis using 126 conservative orthologs (Figure 1). The operation taxonomic units were clearly divided into two clades (bootstrap percentage [BP] = 100): clade A (NIES-39, NIES-46, Paraca, and YZ) and clade B (O9.13F, PCC8005, TJSD091, TJSD092, UTEX-2342, and PCC9438). These clades may correspond to two previously reported clusters determined via rDNA restriction analysis 20 and phylogenetic analysis using the 16S rRNA and *cpcBA-*IGS sequences 21. In clade A, NIES-46 was a robust sister of NIES-39 (BP = 100). This observation is surprising because the sampling locations of NIES-46 (Mexico) and NIES-39 (Chad) are separated by the Atlantic Ocean. Although the distribution pattern remains unknown, the strain might be migrated via wind, birds 22,23, or human activities (e.g., through fish stocking) 24. To elucidate the nucleotide-level similarity between NIES-46 and NIES-39, we performed BLASTn analyses using transcript sequences from both strains with an e-value cut-off <1E-5. The transcript similarity was 99.8% on average. The genome structures of NIES-46 and NIES-39 were compared via progressiveMauve 25. At least six locally colinear blocks and only two short inversions (9,696 bp and 25,438 bp) were found (Figure S1), contrast to the known high genome plasticity in *Arthrospira* strains, which includes many rearrangements 8. These observations suggest that these strains diverged recently. The putative protein functions among the *Arthrospira* species in clade A were compared based on COG classification 26. The protein functions encoded by the NIES-46 genome are highly similar to those in the genomes of NIES-39 and the Paraca strain (Figure S2). The similarity of the NIES-46 and NIES-39 genomes is consistent with their shared phenotypic features, including their similar growth conditions 27. In particular, we focused on genes involved in hydrogen production, including *hoxEFUHY* and *hypABCDEF*. The NIES-46 genome encodes all of these genes, whose loci are conserved among *Arthrospira* strains, suggesting that *Arthrospira* strains have a conserved hydrogen production ability. Recently, an effective transformation system for *A. platensis* has been developed using a type 1 restriction inhibitor and liposomes to protect the exogenous DNA 28, and the *hox* and *hyp* loci could be future targets for transformation to increase hydrogen productivity.

The NIES-46 genome sequence and its annotation should be useful tools for bioengineering applications, including the generation of mutant strains with increased hydrogen production; furthermore, this study provides genomic insight into the dispersion of *Arthrospira* species. Because the natural habitats of NIES-46 and NIES-39, Lake Texcoco 29 and Lake Chad 30, respectively, are shrinking, these strains should be preserved in multiple culture collections to sustain *Arthrospira* genetic diversity.

## Supplementary Material

Supplementary figures.

## Figures and Tables

**Table 1:** *A. platensis* NIES-46 genome features

Feature	Characteristic
Assembly length	5,728,646 bp
Number of contigs	343
N50	153 kbp
GC content	44.50%
Number of protein-coding genes	5,008
Number of rRNAs	4
Number of tRNAs	38
Number of CRISPRs	8

## Abbreviations

BP: bootstrap percentage
