# Supplementary material for: The Draft Genome of a Hydrogen-producing Cyanobacterium, Arthrospira platensis NIES-46
Source: J Genomics. 2019 Sep 18;7:56–9. doi: 10.7150/jgen.38149 (PMC6775862; doi:10.7150/jgen.38149)

The draft genome of a hydrogen-producing cyanobacterium, *Arthrospira platensis* NIES-46

Shigekatsu Suzuki*, Haruyo Yamaguchi, and Masanobu Kawachi

Center for Biology and Environmental Studies, National Institute for Environmental Studies, 16-2 Onogawa, Tsukuba, Ibaraki, Japan

*Corresponding author (S. Suzuki): e-mail address: suzuki.shigekatsu@nies.go.jp; postal address: 16-2 Onogawa, Tsukuba, Ibaraki 305-8506 Japan

Figure S1. Mauve alignment of the NIES-46 and NIES-39 genomes.

ProgressiveMauve was performed with the default options using the NIES-39 genome as a reference. The NIES-39 (above) and NIES-46 (below) genomes are shown. Corresponding colinear blocks are linked by lines. The red vertical bars in the NIES-46 genome indicate the contigs.

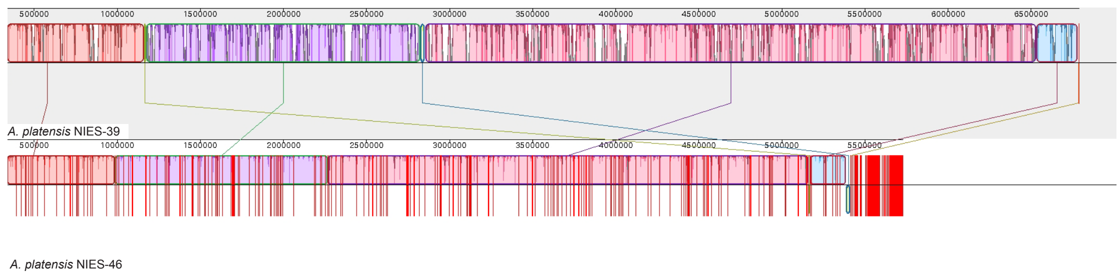

Figure S2. Gene content of the clade A *Arthrospira* species.

The functional categories are based on COG classification. The heatmaps represent the number of genes.

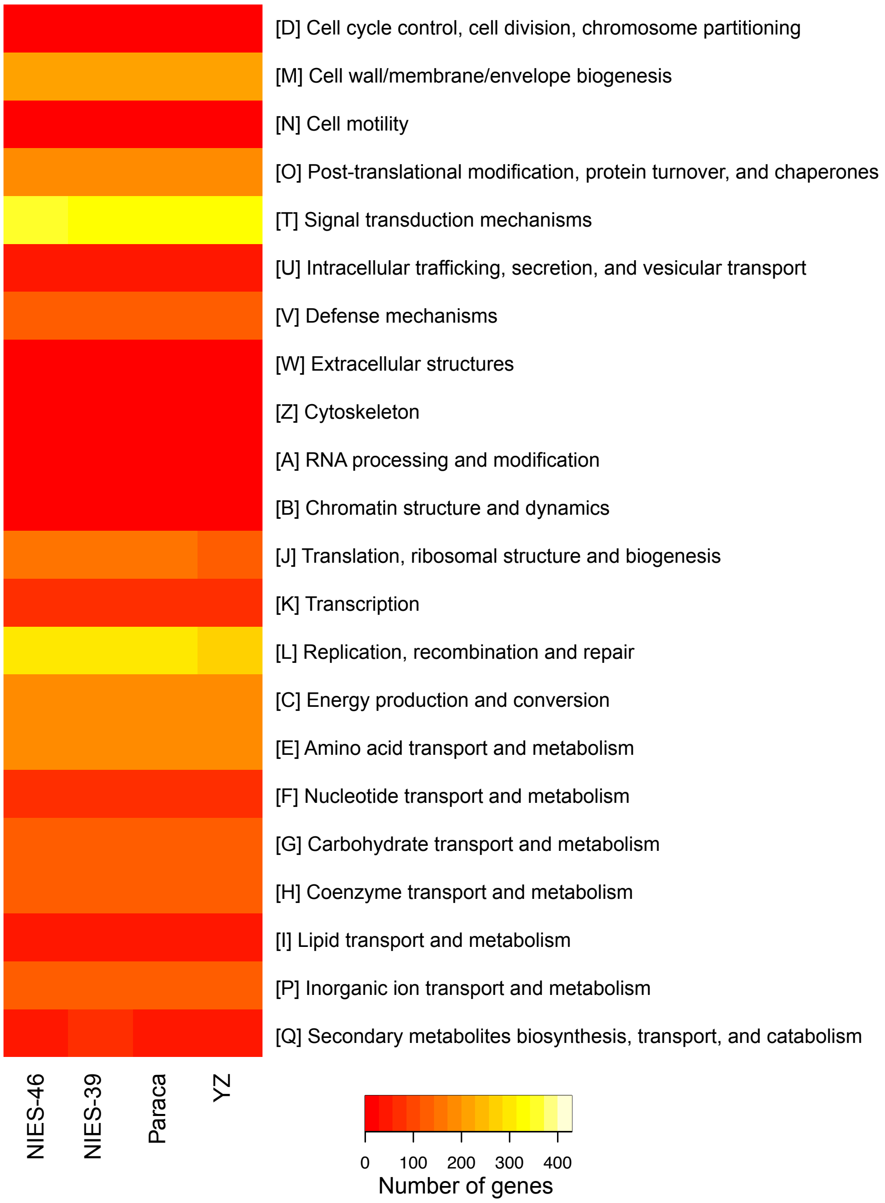

Supplement: Supplementary file 1 — Supplementary figures. [file jgenv07p0056s1.pdf]
